# Association of Dietary Pattern, Lifestyle and Chronotype with Metabolic Syndrome in Elderly—Lessons from the Population-Based Hamburg City Health Study

**DOI:** 10.3390/ijerph19010377

**Published:** 2021-12-30

**Authors:** Claudia Terschüren, Lukas Damerau, Elina Larissa Petersen, Volker Harth, Matthias Augustin, Birgit-Christiane Zyriax

**Affiliations:** 1 Institute for Occupational and Maritime Medicine (ZfAM), University Medical Center Hamburg-Eppendorf (UKE), 20459 Hamburg, Germany; l.damerau@uke.de (L.D.); harth@uke.de (V.H.); 2 Department of Cardiology, University Heart and Vascular Center, 20251 Hamburg, Germany; e.petersen@uke.de; 3 Population Health Research Department, University Heart and Vascular Center, 20251 Hamburg, Germany; 4 Competence Center for Health Services Research in Vascular Diseases (CVvasc), Institute for Health Service Research in Dermatology and Nursing (IVDP), University Medical Centre Hamburg-Eppendorf (UKE), 20246 Hamburg, Germany; m.augustin@uke.de; 5 Midwifery Science—Health Services Research and Prevention, Institute for Health Service Research in Dermatology and Nursing (IVDP), University Medical Centre Hamburg-Eppendorf (UKE), 20246 Hamburg, Germany

**Keywords:** metabolic syndrome, chronotype, diet, DASH, cohort, elderly

## Abstract

In aging populations, the increasing prevalence of metabolic syndrome and the resulting elevated risk of developing non-communicable diseases is a major challenge for worldwide health care. The elderly population-based Hamburg City Health Study (HCHS) allows investigating the association in the relevant age group 45–74 years. For 3513 of 10,000 participants, self-reported information on dietary patterns (DASH, MEDAS), chronotype, lifestyle, and data on metabolic syndrome parameters was available. Overall, having a “low” DASH score was a statistically significant risk factor (OR 1.23; 95% CI 1.01–1.48). Only for “late” chronotype, a slightly elevated OR (1.06) was found, but no statistically significant effect on the outcome of metabolic syndrome. Still, considering chronotype vastly improved the model. However, a trend of an increasing predicted probability from early to late chronotype was found for participants with low adherence to the DASH diet. Future research should focus on options for prevention in persons with late chronotype, so they can be supported better in adherence to, e.g., DASH diet to lower their risk.

## 1. Introduction

The metabolic syndrome (MetS) is associated with a higher risk of non-communicable diseases like nonalcoholic fatty liver disease (NAFLD), cardiovascular disease (CVD), and several types of cancer. According to the International Diabetes Federation (IDF), MetS is defined as a cluster of abdominal adiposity, glucose intolerance, increased blood pressure, and dyslipidemia [1]. Due to the increased number of comorbidities and physiological changes that develop at older ages—such as an increase in the percentage of body fat and decrease in muscle mass [2], older adults are particularly at risk of developing MetS.

In Germany, the Study of Health in Pomerania (SHIP) showed an overall prevalence of 23.8% (women: 18.6%; men: 29.1%) of MetS in the adult population [3]. In the youngest age group (20 to 29 years), the prevalence was 4.0% in women and 6.6% in men, whereas in the age group of 70 years and older, 44.7% of the women and 39.8% of the men met the criteria for MetS. Referring to these results, almost half of the older population is developing MetS.

In Germany, trends by age and educational status (ES) are observed for single parameters that compose the MetS. The German nationwide survey GEDA 2014/2015-EHIS showed a 12-month-prevalence of diabetes mellitus type 2 of 7.7% in adults (18 years and older) [4]. In women, the prevalence was lower (7.0%) than in men (8.6%), based on 23,345 participants [4]. For women aged 45 to 64 years of age, a prevalence of 5.2% (men: 9.3%) was found; in women older than 65 years, a prevalence of 17.6% (men: 21.1%) was observed. Women older than 65 with a low ES more often reported type 2 diabetes (12-month-prevalence: 20.5%) compared to women of the same age with a high ES (15.9%). In men, a similar difference was observed (ES low: 24.0%; ES high: 19.4%). The same trends were found for increased blood pressure in the GEDA survey 2014/2015 and for dyslipidemia in the DEGS 2010 [5]. The 12-month-prevalence of high blood pressure increased by age (45 to 65: women 31.6%; men 38.6% vs. 65 older: women 63.8%; men 65.1%) and differed by ES (high: women 58.0%; men 64.5% vs. low ES: women 66.4%; men 65.5%). As to dyslipidemia, the 12-month-prevalence increased by age and particularly in women (45 to 65: women: 21.5.7%; men: 27.2% vs. 65 older: women 41.2%; men 39.5%). The age effect is even stronger when considering the ES.

In light of the demographic changes and deficits in health literacy, better prevention concepts concerning healthy aging are highly important. Dietary habits and physical activity are well-known factors in the development of MetS [6]. Different aspects of dietary pattern, including energy intake, macronutrient composition, fiber intake, and food pattern, seem to influence the prevalence of MetS and its clustered individual parameters [7]. According to a recent comprehensive review, scientific evidence supports the use of a “Dietary Approaches to Stop Hypertension (DASH)” diet intervention as the paradigm for MetS prevention and treatment [7]. Small dietary changes rather than the restriction of single nutrients are seen to be protective. On top of that, the emerging roles of circadian rhythms and sleep influencing food and nutrient intake should be considered and may act as modifying factors.

Further research is needed to investigate if chronotype modifies the association between food intake, dietary patterns, and MetS [8]. Suh et al. (2017) [9] found that older adults with an evening chronotype consume more caffeinated beverages plus eat heavier meals at night before sleeping. Other studies found that evening chronotypes showed a lower adherence toward a healthy Nordic diet, were more often physically inactive, and reported lower perceived health [10]. In the National FINRISK 2007 Study population, participants who were evening type consumed more fat and less vitamin D, and they showed a higher intake of alcohol and sucrose [11]. Patterson et al. (2016) observed similar habits in late chronotypes. They consumed, on average, fewer daily servings of fruits and vegetables than other early or intermediate chronotypes [12]. The recent scoping review of Mazri et al. (2020) on chronotype and dietary patterns among adults confirms these findings for evening-type individuals based on an evaluation of 29 studies, mostly executed in a cross-sectional design [13].

A recent review of Almoosawi et al. (2019) stated that chronotype might modulate physiologic processes linked to cardiovascular health, including heart rate, blood pressure, and blood lipid concentrations. The authors also reason that chronotype may affect dietary intake and eating patterns, which might result in disturbed glucose metabolism, and in the long term, promotes type 2 diabetes and MetS [8]. Roenneberg et al. (2012) found that sleep duration and living against the inner clock increases BMI in overweight persons [14]. In older age, during retirement, sleeping habits on weekdays and free weekends became more alike, and chronotype in average changes into more early types.

The population-based Hamburg City Health Study (HCHS) focuses on the age group 45 to 74 years [15]. Thus, this mid-aged and elderly study population allows one to investigate the association of diet, eating pattern, chronotype, lifestyle, and MetS in the relevant age group. The MetS emerges predominantly and consolidates in the persons affected. With this analysis, we want to investigate if taking the chronotype into account might provide a possible approach in the prevention of MetS and in reducing sequelae like stroke, infarction, or diabetes mellitus type 2 in the elderly.

## 2. Materials and Methods

### 2.1. Study Population

This study is embedded in the ongoing “Hamburg City Health Study“ (HCHS). The HCHS aims to build a cohort of finally 45,000 population-based participants in Hamburg (Jagodzinski et al., 2020) [15]. Eligible were individuals from 45 to 74 years of age at time of sampling and lived in Hamburg, Germany. Potential participants were randomly invited via the registration office. They received a letter with the data protection declaration and the first questionnaire. Later, they were invited to the University Medical Center Hamburg-Eppendorf (UKE) if their language skills were sufficient to comprehend the questionnaires and instructions from the study personnel and able to participate in the medical examinations. All participants gave written informed consent. Participants’ data of this study were extracted from the dataset of the first sub-cohort HCHS (*n* = 10,000). Data for this sub-cohort was collected between 2016 and 2019.

For this analysis, needed data was available for 4330 of 10,000 participants. Those with a prior history of heart attacks, strokes, and illnesses associated with the coronary vessels were excluded (*n* = 358) due to the potential confounder risk in association with the outcome variable MetS.

Margin age groups, which were disproportionally underrepresented in HCHS compared to the reference population of the City of Hamburg, were excluded to reduce bias. The final sample size enclosed in this analysis amounts to *n* = 3513.

### 2.2. Outcome Variable—MetS

The outcome MetS was based on the definition of the IDF [1]. Height, weight, waist circumference, and blood pressure were measured by trained study nurses at the HCHS study center, and blood samples were taken. Participants were asked to bring their medication packages, so their medication’s specific product codes (PZN, “Pharmazentralnummer”, a unique German barcode identifier for medicinal products) could be scanned. That allowed to determine whether the participants used a drug associated with parameters of the MetS.

To assign the status of MetS according to IDF, participants had to be abdominal adipose (waist circumference of >94 cm in men and of >80 cm in women). Additionally, at least 2 of the 4 following conditions must be fulfilled:

1. Raised triglycerides ≥ 150 mg/dL or specific medication for this lipid abnormality.
2. Reduced HDL cholesterol (men < 40 mg/dL, women < 50 mg/dL) or specific medication for this lipid abnormality/
3. Raised blood pressure exceeds ≥ 130 mm Hg systolic or ≥ 85 mm Hg diastolic or medication of the previously diagnosed hypertension.
4. Raised fasting plasma glucose ≥ 100 mg/dL or previously diagnosed type 2 diabetes mellitus.

### 2.3. Dietary Scores

Information on dietary intake was collected by validated questionnaires developed for the European Prospective Investigation into Cancer and Nutrition (EPIC) study [16]. The used dietary questionnaire (food frequency questionnaire, version 2 (FFQ2)) records the frequency and portion size of 102 food items eaten during the previous year [16]. Relevant food groups, energy intake, and nutrients were assessed.

Dietary Approaches to Stop Hypertension (DASH) To calculate adherence to the DASH diet, a scoring scheme from Folsom et al. (2007) was used [17]. The overall score included 10 equally weighted food items (frequency consumption of grains, vegetables, fruits, dairy, meat/poultry/fish, nuts/seeds/legumes, sweets obtained from raw data) and average daily intakes of certain nutrients (fat, saturated fat, sodium) (Table 1). A score of 0–1 was assigned for each dietary component and summed across the 10 items [18]. A score of 10 reflected full adherence, while 0 represented non-adherence.

Mediterranean Diet Adherence Screener (MEDAS) To assess adherence to the Mediterranean diet, the validated German translation of the original MEDAS was used [19]. The MEDAS score is based on 12 questions on food items (frequency consumption of food groups by using raw data, mean daily intake of vegetable oil and animal fat) and 2 questions on food intake habits characteristic of the Mediterranean diet.

Each item was scored with a 0 (condition not met) or a 1 (adherent); thus, the MEDAS score ranged from 0 to 14 points (Appendix A Table A1). The MEDAS scores of the participants were then transformed into tertiles, resulting in three equally sized groups as ”low”, “medium”, and “high”.

### 2.4. Chronotype

Individual sleeping habits were ascertained based on the “Munich Chronotype Questionnaire” [20]. The chronotype was calculated for work-free days. Exclusion criteria were the use of alarm clock, dependent sleep cycle (due to, e.g., care for family members), implausible total sleep duration of fewer than 3 h or more than 15 h, or missing values in sleep onset or wake-up time. Due to quality assurance measures, implausible values (*n* = 100) were corrected, e.g., if persons obviously mixed up the 24-h system and time in a.m./p.m. “Normal” chronotypes had rounded to half an hour mid-sleeps between 04:00 and 04:30, early between 03:30 and 23:00, and late between 05:00 and 10:00.

### 2.5. Covariates

To assess physical activity, participants completed the European Prospective Investigation into Cancer and Nutrition Physical Activity Questionnaire (EPIC-PAQ) [21]. The activity was assessed separately for summer and winter. Time was cumulated and divided by two to receive a proxy for the year’s weekly average in total. This was transferred into a variable dichotomizing equal to or more than 1 h/week (reference) and less than 1 h/week.

The smoking status was queried during the anamnesis at the study center. The analysis included smoking status using three levels: never smoker, ex-smoker for at least six months, and current smoker.

As a proxy for socioeconomic status, the highest level of school-leaving qualification was included in data analysis (divided into three groups “high”, “medium,” and “low”).

### 2.6. Statistical Analyses

Binary logistic regression was used to determine the risk factors of the independent variables age, sex, sport, educational level, smoking status, and DASH diet to the dependent outcome variable: MetS (risk estimates with a 95% confidence interval). Each variable in the regression model was tested for interaction. A *p*-value of <0.05 was considered statistically significant. Statistical analyses were done using R 3.6.2 (R Development Core Team, Auckland, New Zealand).

## 3. Results

### 3.1. Study Sample

The final sample size consisted of *n* = 3513 complete-case participants. In Table 2, the characteristics of the participants are shown (women: 50.4%, men: 49.6%). The average age was 61.4 years (SD 6.8, range 50 to 74). Men reported more often a high education level (“high”) according to their school diploma (high education: 48.3% in women, 58.5% in men). Women and men almost equally often were “current-smoker,” but women reported more often to be “never-smoker” (women: 40.1% vs. men: 33.7%).

More women were categorized as “normal” chronotype (women: 45.6% vs. men: 42.5%). Men more often showed a “late” chronotype (women 25.4% vs. men 29.0%).

On average, women had a higher DASH score. Men were twice as often assigned, the “low” category of the DASH score tertile as women. The same trends applied to the MEDAS score.

Participants classified as an “early” chronotype showed the lowest daily calorie intake, and persons categorized as “late” chronotype the highest (see Table 3). “Late” chronotype participants showed a statistically significant lower DASH score than the other two chronotype groups. “Late” chronotype also received a “high” DASH score less often. This was not observed for the MEDAS score.

Women were more often adipose according to the measured waist circumference (women: 73.9% vs. men: 69.2%, see Table 4). Men had worse metabolic index parameters, apart from the reduced HDL, which showed no sex difference. MetS (IDF) showed 28.2% of women and 44.2% of men.

### 3.2. Results of Logistic Regression Analyses

Table 5 shows the logistic regression model results, including all 3513 participants. Risk factors for MetS were higher age, male sex, less than 1 h of sport per week, and not having a “high” school diploma. Having a “low” DASH score was a statistically significant risk factor (OR 1.23; 95% CI 1.01–1.48). Low and medium MEDAS scores were risk factors, but none of the odds ratios statistically reached significance. Only for “late” chronotype, a slightly elevated OR (1.06) was found, but no statistically significant effect on the outcome MetS, but improved the model.

**Figure 1:**
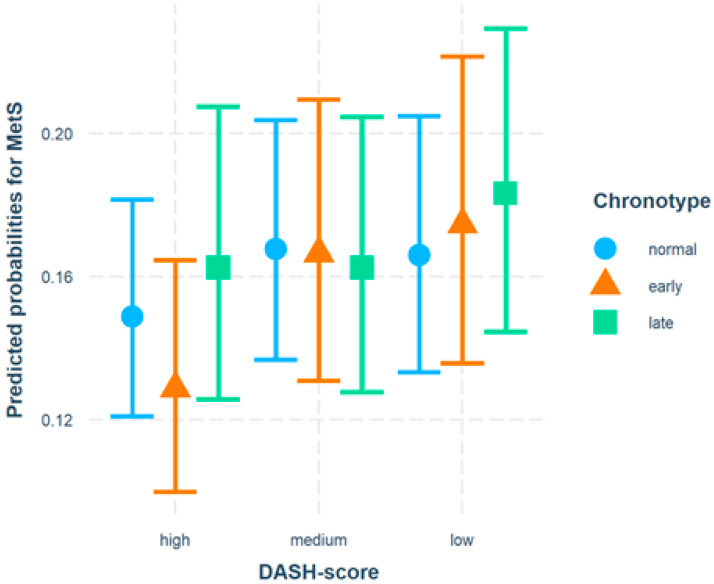
Multiple logistic regression with an interaction term between DASH score and chronotype with metabolic syndrome (MetS) as a dependent variable. Adjusted for age, sex, sport, school diploma, and smoking status (*n* = 3513).

Figure 1 displays the test for possible interaction between DASH and chronotype. Whereas within the “low” DASH score category, a trend of increasing risk of MetS from normal via early to late chronotype can be observed, the categories of high and moderate adherence to DASH diet showed no trend.

## 4. Discussion

In this cross-sectional study, we investigated the association between chronotype, dietary quality, and the prevalence of the MetS in an elderly population-based cohort, considering age, sex, education, and further relevant lifestyle factors. Overall, we found that women had a higher DASH score, whereas men were twice as often assigned the “low” DASH category. The same was found in an analysis of periodontitis [22]. Higher adherence to the DASH or the MEDAS diet was associated with less conventional cardiovascular risk factors and the prevalence of MetS. Participants assigned to the “late” chronotype were more often characterized by higher energy intake and a lower DASH score. In a logistic regression analysis, age, male sex, less than 1 h of sport per week, not having a high school diploma, and being categorized to the low DASH score were independent risk factors for the MetS, while the chronotype vastly improved the model but failed to show significance.

In this elderly population from the HCHS study, the prevalence of MetS was higher in men than women (men: 44.2% vs. women: 28.2%, see Table 4). Our observation that males were particularly characterized by a cluster of metabolic risk factors, including higher blood pressure values, impaired glucose metabolism, and raised triglycerides is consistent with German nationwide research [4,5]. However, more women than men presented with central obesity despite lower mean BMI. This finding can be explained predominantly by hormonal and metabolic changes that occur during the menopause transition. Actually, estrogen loss adversely alters body fat distribution, cholesterol profile, blood pressure, and increased insulin resistance [23].

Central obesity independent of BMI is the key factor in the development of MetS. Besides hereditable determinants, modifying lifestyle factors such as dietary habits, physical activity, and to a certain extent, smoking status, remain the major driving force [24]). Overall, elderly women in HCHS followed a favorable lifestyle compared to men. Significantly more women described themselves as never smokers, as physically active (≥1 h sports/week), and reported higher adherence to both healthy dietary patterns, DASH and MEDAS. More than twice as many women as men were assigned to the highest tertile of DASH and the MEDAS score. Sex-specific differences in health behavior, particularly dietary habits and related risk factors, are well-known among adult populations [25,26]. Although mean adherence to the DASH score was underparts (4.48 of 10) in the study population, it was significantly higher in the older age group (65–74 yr) compared to younger participants (50–64 yr). A healthier food choice with increasing age has been described elsewhere [27].

As expected from previous research, adherence to the DASH diet seemed to be cardio-protective [7,23]. The prevalence of conventional cardiovascular risk factors was slightly but significantly lower in the highest tertile compared to the lowest. This includes central obesity (68.6% vs. 71.6%), elevated blood pressure (56% vs. 64.9%), diabetes (21.7% vs. 29.6%), raised triglycerides (16.4% vs. 28%) and low HDL-cholesterol (8.1% vs. 13.4%). The same trend applied to the MEDAS diet. Consequently, high adherence to both diets was associated with a lower prevalence of MetS (DASH: 29.6% vs. 42.1%; MEDAS: 29.2% vs. 41.7%).

In this analysis, individual chronotype was referable for 3513 participants (women: *n* = 1772; men: 1741). Chronotype was classified as “normal” in 44.1% of the participants, “early” in 28.8%, and “late” in 27.2%. Almost equally in men and women, one-third was classified as “early” chronotype (28.5% vs. 29.0%). More men than women were classified as “late” chronotype (men: 29.0% vs. women: 25.4%). This finding is consistent with a meta-analysis of Randler and Engelke (2019), which included 164 studies. Randler and Engelke also observed that gender differences diminish with age, but still, older women were less often early chronotype than older men [28]—a result not found in our sample.

Growing evidence indicates the contribution of chronotype to central obesity [24]. A scoping review of Mazri et al. (2020) concluded that the late chronotype is associated with a delay in meal timing, skipping breakfast, and more meals during nighttime, together with higher consumption of sweets, caffeine, and alcohol [13]. Morning and evening types were relatively alike in consuming carbohydrates, fat, cholesterol, fiber, legumes, meat, fish, or dairy products. Chronotype has often been linked to dietary intake. Individuals with “late” chronotype particularly follow an unhealthier dietary behavior [10,13,23,29]. These studies are largely supportive of our findings. Participants classified as “late” chronotypes showed the highest energy intake, statistically significant, compared to those with a “normal” chronotype. Additionally, “late” chronotypes belong less often to the higher DASH score category and were characterized by a significantly lower DASH score in contrast to the other two chronotypes. Differences between chronotype and the MEDAS score failed to show significance. The lack of association is likely due to the overall low adherence to the MEDAS score (mean: 4.54 points out of max possible 14 points) in this aging, non-Mediterranean population. For example, less than 5% of the participants fulfilled key diet components, such as the intake of olive oil, fish/seafood, red meat and pulses. Thus, even those in the highest tertile did not eat in accordance with the recommendations.

Depending on the completeness of data to classify chronotype, 3513 participants were included in the multiple logistic regression model. As expected being male (OR = 1.93; 95% CI 1.93–2.25), 65 years or older (OR = 1.54; 95% CI 1.32–1.79) and a low educational level (OR = 2.04; 95% CI 1.68–2.49) stand for statistically significant elevated risks for MetS.

Physical activity is known to be important in the prevention and as an intervention for MetS [6,30]. According to this, our analysis showed “less than 1 h of sport per week” as a risk factor (OR = 1.55; 95% CI 1.32–1.81).

Less impact was found for the DASH diet in this elderly cohort. Only for participants with low adherence, the elevated risk of MetS became statistically significant (OR = 1.23; 95% CI 1.01–1.48). In a review of Filippou et al. (2020), it was found that in trials investigating the DASH diet with mean age < 50-years, blood pressure was reduced more, so our findings are plausible in this context [31].

Chronotype was not directly associated with MetS, neither “early” nor “late.” A slightly elevated OR (1.06) was observed for late chronotype but not statistically significant. That was why we tested for a possible interaction between the DASH diet and chronotype (Figure 1). For participants with low adherence to the DASH diet, a trend of an increasing predicted probability from early to late chronotype was found. Such a trend was not clear in the DASH categories with medium or high scores. It might be concluded that a combination of late chronotype and low DASH score possibly increased the risk of developing a MetS.

Our findings will add to the limited number of studies investigating the association between chronotype, dietary quality, and MetS in an elderly population-based cohort, considering age, sex, and education as well as physical activity and smoking status. Chronotype and dietary and lifestyle habits were assessed by validated methods/instruments. However, a cross-sectional design can not reveal causality, and information on chronotype, diet, and lifestyle behavior were based only on self-reported data. In this data analysis based on the first 10,000 participants, health-conscious individuals were more likely to participate in the investigation, which we observed referring to the level of education and proportion of current smokers or adipose persons [32], respectively. Therefore, reporting and selection bias can not be excluded.

## 5. Conclusions

In total, the data showed that in HCHS, known risk factors for MetS are confirmed. However, lifestyle factors like smoking or lack of exercise are contributing less than low education.

Participants of this northern German HCHS cohort investigated here are quite similar in diet adherence related to the prevention of hypertension. Men and women score close around four underparts with a small standard deviation for DASH adherence. But only those attributed to the lowest tertile of the DASH score we observed to be at risk of MetS. Within this group, late chronotype was found to be an unfavorable condition.

Future research should focus on options for prevention in persons with late chronotype, so they can be supported better in adherence to, e.g., DASH diet to lower their risk.

## Figures and Tables

**Table 1:** Scoring criteria for DASH Dietary Adherence.

Score Items	DASH Component	Scoring
1	Total Grain	
	≥7 servings/day	1
	5–6 servings/day	0.5
	<5 servings/day	0
2	Vegetables	
	≥4 servings/day	1
	2–3 servings/day	0.5
	<2 servings/day	0
3	Fruits	
	≥4 servings/day	1
	2–3 servings/day	0.5
	<2 servings/day	0
4	Total dairy	
	≥2 servings/day	1
	1 servings/day	0.5
	<1 serving/day	0
5	Meat, poultry, and fish	
	≤2 servings/day	1
	3 servings/day	0.5
	≥4 serving/day	0
6	Nuts, seeds, and legumes	
	≥4 servings/day	1
	2–3 servings/day	0.5
	<2 servings/day	0
7	% kcal from fat	
	≤27%	1
	≥28 ≤29%	0.5
	≥30%	0
8	% kcal from saturated fat	
	≤6%	1
	≤7 ≥8%	0.5
	≥9%	0
9	Sweets	
	≤5 servings/week	1
	6–7 servings/week	0.5
	≥8 serving/week	0
10	Sodium	
	≤2400 mg/day	1
	2400–3000 mg/day	0.5
	>3000 mg/day	0

**Table 2:** Baseline characteristics of the study population.

	Women	Men	Total
*n* (%)	2070 (47.8%)	2260 (52.2%)	4330 (100%)
Diagnosis heart attack, *n* (%)	19 (0.9%)	101 (4.5%)	120 (2.8%)
Diagnosis stroke, *n* (%)	42 (2.0%)	71 (3.1%)	113 (2.6%)
Diagnosis coronary heart disease, *n* (%)	32 (1.6%)	178 (8.0%)	210 (4.9%)
At least one with diagnosis and thus excluded	81 (100.0%)	277 (100.0%)	358 (100.0%)
Also excluded by age			
45–49 years	153 (7.7%)	137 (6.9%)	290 (7.3%)
75+ years	64 (3.2%)	105 (5.3%)	169 (4.3%)
After exclusion due to prior history of relevant illnesses and age			
*n* (%)	1772 (50.4%)	1741 (49.6%)	3513 (100%)
Age years, mean (SD)	61.1 (6.707)	61.799 (6.8)	61.4 (6.777)
Age categories, *n* (%)			
50–54	368 (20.8%)	326 (18.7%)	694 (19.8%)
55–59	424 (23.9%)	358 (20.6%)	782 (22.3%)
60–64	401 (22.6%)	388 (22.3%)	789 (22.5%)
65–69	341 (19.2%)	399 (22.9%)	740 (21.1%)
70–74	238 (13.4%)	270 (15.5%)	508 (14.5%)
Age ≥ 65 years, *n* (%)	579 (32.7%)	669 (38.4%)	1248 (35.5%)
Sport ≥ 1 h/week, *n* (%)	1326 (74.8%)	1165 (66.9%)	2491 (70.9%)
School diploma, *n* (%)			
low	331 (18.7%)	315 (18.1%)	646 (18.4%)
medium	586 (33.1%)	408 (23.4%)	994 (28.3%)
high	855 (48.3%)	1018 (58.5%)	1873 (53.3%)
Smoking status, *n* (%)			
Never-smoker	711 (40.1%)	587 (33.7%)	1298 (36.9%)
Ex-smoker	735 (41.5%)	820 (47.1%)	1555 (44.3%)
Smoker	326 (18.4%)	334 (19.2%)	660 (18.8%)
BMI, mean (SD)	26.0 (4.8)	27.0 (4.2)	26.5 (4.5)
BMI category, *n* (%)			
Normalweight (BMI 18.5–24.9)	841 (32.0%)	557 (32.0%)	1398 (39.8%)
Undeweight (BMI < 18.5)	30 (1.7%)	5 (0.3%)	35 (1.0%)
Overweight BMI (25–29.9)	599 (33.8%)	843 (48.4%)	1442 (41.0%)
Adipose (BMI ≥ 30)	302 (17.0%)	336 (19.3%)	638 (18.2%)
Sleeptime in h, *n* (%)			
6–8	999 (56.4%)	1024 (58.8%)	2023 (57.6%)
<6	141 (8.0%)	115 (6.6%)	256 (7.3%)
>8	632 (35.7%)	602 (34.6%)	1234 (35.1%)
Chorotype, *n* (%)			
“normal”	808 (45.6%)	740 (42.5%)	1548 (44.1%)
early	514 (29.0%)	496 (28.5%)	1010 (28.8%)
late	450 (25.4%)	505 (29.0%)	955 (27.2%)
Energy intake kcal/day, mean (SD)	1843.4 (572.5)	2518.9 (808.5)	2178.2 (776.7)
DASH score (max. possible 10), mean (SD)	4.82 (0.9)	4.13 (1.0)	4.48 (1.0)
DASH score in tertiles, *n* (%)			
low	366 (20.7%)	797 (45.8%)	1163 (33.1%)
medium	620 (35.0%)	586 (33.7%)	1206 (34.3%)
high	786 (44.4%)	358 (20.6%)	1144 (32.6%)
MEDAS score (max. possible 14), mean (SD)	5.16 (1.7)	3.91 (1.7)	4.54 (1.8)
MEDAS score in tertiles, *n* (%)			
low	357 (20.1%)	827 (47.5%)	1184 (33.7%)
medium	606 (34.2%)	563 (32.3%)	1169 (33.3%)
high	809 (45.7%)	351 (20.2%)	1160 (33.0%)

**Table 3:** Energy and dietary adherence score characteristics stratified after Chorotype.

	“Normal” (*n* = 1548)	Early (*n* = 1010)	Late (*n* = 995)	Total (*n* = 3513)	*p*-Value “ Normal” vs. Early	*p*-Value “ Normal” vs. Late
Energy intake, kcal/day, mean (SD)	2179.5 (784.1)	2101.0 (719.8)	2257.8 (814.2)	2178.2 (776.7)	0.024 (1)	0.009 (1)
DASH score, mean (SD)	4.53 (1.1)	4.50 (1.0)	4.37 (1.1)	4.48 (1.0)	0.552 (1)	<0.001 (1)
DASH score in tertiles, *n* (%)					0.900 (2)	0.004 (2)
- low	485 (31.3%)	325 (32.2%)	353 (37.0%)	1163 (33.1%)		
- medium	533 (34.4%)	345 (34.2%)	328 (34.3%)	1206 (34.3%)		
- high	530 (34.2%)	340 (33.7%)	274 (28.7%)	1144 (32.6%)		
MEDAS score, mean (SD)	4.56 (1.9)	4.52 (1.8)	4.53 (1.8)	4.54 (1.8)	0.412 (1)	0.492 (1)
MEDAS score in tertiles, *n* (%)					0.248 (2)	0.475 (2)
- low	500 (32.3%)	356 (35.2%)	328 (34.3%)	1184 (33.7%)		
- medium	517 (33.4%)	333 (33.0%)	319 (33.4%)	1169 (33.3%)		
- high	531 (34.3%)	321 (31.8%)	308 (32.3%)	1160 (33.0%)		

(1) Kruskal-Wallis rank-sum test; (2) Pearson’s Chi-squared test.

**Table 4:** Characteristics of the metabolic syndrome, according to the IDF index (2005).

	Women (*n* = 1772)	Men (*n* = 1741)	Total (*n* = 3513)	*p*-Value
Waist circumference (>80 cm women, >94 cm men), *n* (%)	1298 (73.9%)	1198 (69.2%)	2496 (71.6%)	0.002 (1)
Triglycerides > 150 mg, *n* (%)	300 (17.0%)	476 (27.5%)	776 (22.2%)	<0.001 (1)
HDL (<50 mg/dL women, <40 men), *n* (%)	187 (10.6%)	200 (11.5%)	387 (11.1%)	0.369 (1)
Blood pressure (systolic > 130 mmHg or diastolic > 85 mmHg), *n* (%)	924 (53.4%)	1145 (67.3%)	2069 (60.3%)	<0.001 (1)
Fasting plasma glucose (>100 mg/dL) or diabetes mellitus, *n* (%)	305 (18.4%)	541 (34.1%)	846 (26.1%)	<0.001 (1)
Metabolic syndrome, *n* (%)	500 (28.2%)	770 (44.2%)	1270 (36.2%)	<0.001 (1)

(1) Pearson’s Chi-squared test.

**Table 5:** Multiple logistic regression. Adjusted odds ratio (OR) estimates of pre-selected independent variables to the dependent variable metabolic syndrome (*n* = 3513).

Risk Factor	Category	Reference	OR	95% CI	*p*-Value
Age	≥65	<65	1.54	(1.32, 1.79)	<0.001
Sex	men	women	1.93	(1.65, 2.25)	<0.001
School diploma	medium	high	1.48	(1.24, 1.75)	<0.001
	low		2.04	(1.68, 2.49)	<0.001
Smoking status	ex-smoker	never-smoker	1.29	(1.10, 1.52)	0.002
	current smoker		1.47	(1.20, 1.81)	<0.001
Sport/physical training	<1 h/week	≥1 h/week	1.55	(1.32, 1.81)	<0.001
Chronotype	early	“normal”	0.97	(0.81, 1.15)	0.698
	late		1.06	(0.89, 1.26)	0.522
DASH score (tertiles)	medium	high	1.16	(0.97, 1.40)	0.100
	low		1.23	(1.01, 1.48)	0.035

## Data Availability

Due to the nature of this research, participants of this study did not agree for their data to be shared publicly, so supporting data is not available.

## Appendix A

**Table A1:** Scoring criteria for Mediterranean Dietary score.

Score Item	MEDAS Question	Data Recorded by FFQ 1 Point Given, if …
1	Do you use olive oil as the principal source of fat for cooking?	use of olive oil for the preparation of at least 2 of the following groceries: salad, vegetable, meat/fish
2	How much olive oil do you consume per day (including that used in frying, salads, meals eaten away from home, etc.)?	based on FFQ calculation, if >48 g vegetable oil per day
3	How many servings of vegetables do you consume per day?	based on FFQ calculation, if ≥2 portions of vegetables per day (including raw and cooked vegetables, salad, olives, mushrooms except potatoes and legumes)
4	How many pieces of fruit (including fresh-squeezed juice) do you consume per day?	based on FFQ calculation, if ≥3 portions of fruit (including fruit, mixed fruit, fruit salad, mixed stewed fruit and fruit juices excluding sweetened beverages)
5	How many servings of red meat, hamburger, or sausages do you consume per day?	based on FFQ calculation, if <100 g red meat (eg beef, veal, pork, lamb) and processed meat products
6	How many servings (12 g) of butter, margarine, or cream do you consume per day?	based on FFQ calculation, if <1 portion butter, margarine and cream and other animal fat
7	How many carbonated and/or sugar-sweetened beverages do you consume per day?	based on FFQ calculation, sugar-sweetened beverages <1 portion per day (including lemonade and colas)
8	Do you drink wine? How much do you consume per week?	based on FFQ calculation, if ≥7 portions wine (red and white wine; 1 portion = 0.251)
9	How many servings of pulses do you consume per week?	≥3 portions pulses (e.g., beans, lentils, peas, chickpeas)
10	How many servings of fish/seafood do you consume per week?	based on FFQ calculation, if ≥3 portions fish, fish products and seafood per week
11	How many times do you consume commercial (not homemade)pastry such as cookies or cake per week?	based on FFQ calculation, if <3 portions cakes, chocolate, cookies, sweets with and without chocolate per week
12	How many times do you consume nuts per week?	based on FFQ calculation, if ≥3 portions nuts per week
13	Do you prefer to eat chicken, turkey or rabbit instead of beef, pork, hamburgers, or sausages?	Based on FFQ calculation, if g white meat (e.g., chicken, hen and other poultry) > g red meat (e.g., beef, veal, pork, lamb and processed meat products)
14	How many times per week do you consume boiled vegetables, pasta, rice, or other dishes with a sauce of tomato, garlic, onion, or leeks sautéed in olive oil?	>1–2 times a week tomato sauce
